# Occurrence and characterisation of biofilms in drinking water systems of broiler houses

**DOI:** 10.1186/s12866-019-1451-5

**Published:** 2019-04-15

**Authors:** Sharon Maes, Thijs Vackier, Son Nguyen Huu, Marc Heyndrickx, Hans Steenackers, Imca Sampers, Katleen Raes, Alex Verplaetse, Koen De Reu

**Affiliations:** 1 Flanders Research Institute for Agriculture, Fisheries and Food (ILVO), Technology and Food Science Unit, Brusselsesteenweg 370, 9090 Melle, Belgium; 2 Faculty of Engineering Technology, Department of Microbial and Molecular Systems (M2S), Cluster for Bioengineering Technology (CBeT), Laboratory of Enzyme, Fermentation and Brewery Technology, University of Leuven, Gebroeders De Smetstraat 1, 9000 Ghent, Belgium; 3 Faculty of Bioscience Engineering, Department of Industrial Biological Sciences, Ghent University Campus Kortrijk, Graaf Karel de Goedelaan 5, 8500 Kortrijk, Belgium; 4 Faculty of Veterinary Medicine, Department of Pathology, Bacteriology and Poultry Diseases, Ghent University, Salisburylaan 133, 9820 Merelbeke, Belgium; 5 Faculty of Bioscience Engineering, Department of Microbial and Molecular Systems (M2S), Centre of Microbial and Plant Genetics (CMPG), University of Leuven, Kasteelpark Arenberg 20 box 2460, 3001 Leuven, Belgium

**Keywords:** Biofilm, Broiler, Drinking water system, *Pseudomonas* spp., *Stenotrophomonas maltophilia*

## Abstract

**Background:**

Water quality in the drinking water system (DWS) plays an important role in the general health and performance of broiler chickens. Conditions in the DWS of broilers are ideal for microbial biofilm formation. Since pathogens might reside within these biofilms, they serve as potential source of waterborne transmission of pathogens to livestock and humans. Knowledge about the presence, importance and composition of biofilms in the DWS of broilers is largely missing. In this study, we therefore aim to monitor the occurrence, and chemically and microbiologically characterise biofilms in the DWS of five broiler farms.

**Results:**

The bacterial load after disinfection in DWSs was assessed by sampling with a flocked swab followed by enumerations of total aerobic flora (TAC) and *Pseudomonas* spp. The dominant flora was identified and their biofilm-forming capacity was evaluated. Also, proteins, carbohydrates and uronic acids were quantified to analyse the presence of extracellular polymeric substances of biofilms. Despite disinfection of the water and the DWS, average TAC was 6.03 ± 1.53 log CFU/20cm^2^. Enumerations for *Pseudomonas* spp. were on average 0.88 log CFU/20cm^2^ lower. The most identified dominant species from TAC were *Stenotrophomonas maltophilia*, *Pseudomonas geniculata* and *Pseudomonas aeruginosa*. However at species level, most of the identified microorganisms were farm specific. Almost all the isolates belonging to the three most abundant species were strong biofilm producers. Overall, 92% of all tested microorganisms were able to form biofilm under lab conditions. Furthermore, 63% of the DWS surfaces appeared to be contaminated with microorganisms combined with at least one of the analysed chemical components, which is indicative for the presence of biofilm.

**Conclusions:**

*Stenotrophomonas maltophilia*, *Pseudomonas geniculata* and *Pseudomonas aeruginosa* are considered as opportunistic pathogens and could consequently be a potential risk for animal health. Additionally, the biofilm-forming capacity of these organisms could promote attachment of other pathogens such as *Campylobacter* spp. and *Salmonella* spp.

**Electronic supplementary material:**

The online version of this article (10.1186/s12866-019-1451-5) contains supplementary material, which is available to authorized users.

## Introduction

Drinking water quality and the drinking water system (DWS) play an important role in the general health and performance of livestock, including broiler chickens [1]. Drinking water for broiler chickens can be contaminated with chemical and microbiological components i.a. through the source or through the animals via the drinking cups. *Campylobacter jejuni*, *E. coli*, *Pseudomonas* spp. and *Salmonella* spp. are microorganisms frequently found in drinking water for broilers [1–5]. Waterborne transmission of pathogens to livestock and humans can occur and thereby cause a potential risk for animal and human health [6, 7].

The number of microorganisms can increase when conditions are favourable or when they attach to or form a biofilm on the inside of the DWS. The combination of a convenient temperature (average temperature of ±25 °C in broiler houses), low flow rates and sufficient nutrients makes the DWS in broiler houses ideal for microbial numbers to increase and biofilms to form [7]. Biofilms are sessile communities of microorganisms, surrounded by a matrix of self-produced extracellular polymeric substances (EPS). *Aeromonas* spp., *E. coli, Pseudomonas* spp. and *Sphingomonas* spp. were previously described as biofilm-forming organisms in water systems of bovine and humans, but also *Salmonella* spp. and *Campylobacter* spp. are capable to form biofilms in poultry environments [6, 8–13]. Biofilm-forming capacities of microorganisms depend on several factors such as growth conditions, contact surface and species or strain type [10, 14–17]. Biofilms do not per se contain pathogens, but they can provide a place that is easy to attach for these kind of cells [18, 19]. The presence and composition of biofilm in the DWS of broilers is still insufficiently known. The water quality on broiler farms is regularly evaluated at the source and sometimes at the end of the drinking lines depending on the type of DWS (open or closed), but along the drinking lines (where the animals actually drink) often no assessment is done [6, 20]. Surfaces on the inside of the DWS of broiler chickens are even less or not sampled.

Disinfection of the water and DWS with oxidisers (for example chlorine or hydrogen peroxide), acids or a combination is often performed between production rounds [7], but does not guarantee the elimination of all the microorganisms present. For poultry, drinking water is preferred for medicine administration because of practical reasons [21]. Microorganisms present in biofilms are protected against disinfection products and medicine by the EPS matrix and by enzymes produced by the microorganisms themselves [22–25]. Moreover, medicines (more specifically carrier substances) and additives (e.g. vitamins) administered by the drinking water can serve as a nutrient source for microorganisms and benefit biofilm formation [6]. On the other hand, animals can be under dosed due to the capture of medicine particles in the biofilm matrix, which can lead to risks for animal health and the development of resistant strains [26, 27]. Concerning the development of resistant strains, biofilms are known as hotspots for plasmid transfer and consequently also for the transmission of resistance genes [28–30].

There is a lack of information concerning the occurrence, importance and composition of biofilms on the inside of the DWS of broiler chickens. Therefore, the aim of this study was to sample the inside of the DWS of broiler houses to assess the occurrence and chemical and microbiological characteristics of biofilms. Subsequently, the dominant bacteria were identified and evaluated for their biofilm-forming capacities in an in vitro biofilm model system.

## Materials and methods

### Sampling on broiler farms

On five different Belgian broiler farms (K1-K5), surfaces on the inside of the DWS were sampled during vacancy, approximately 24 h after the disinfection step. More information about water disinfection that was performed by the farmers during production and DWS disinfection during vacancy is provided in Table 1. Sampling points include the end of the pipes, openings at the height of drinking cups, the inside of pressure regulators and water samples before entering the broiler house (thus without disinfection products). In the period July 2015 – October 2016, each broiler house was sampled once or twice (with a time interval of approximately 1 year) resulting in 85 surface and 7 water samples. The plastic surface area of approximately 20cm^2^ was swabbed using the tip of a flocked swab (Copan, Cat#552C, Brescia, Italy). As the diameter of the drinking line is 2 cm, a depth of 3.5 cm of the inside of the line was sampled to obtain an area of 20cm^2^. Also at the level of openings at the height of the drinking cups and pressure regulators the same area of 20cm^2^ was sampled. After sampling, the nylon tip of the flocked swab was deducted from the breakable plastic applicator and placed in a sterile stomacher bag containing 10 ml of ¼ Ringer’s solution (Oxoid, BR0052, Basingstoke, Hampshire, England). Also blank flocked swabs premoistened with ¼ Ringer’s solution were included in the study as a control for materials and reagents sterility and to check the sampling and enumeration procedures. Water samples were collected after 1 min of water flow in a sterile container (231178, Novolab). Surface and water samples were transported to the lab under cooled conditions. In the lab, sampled surface material in the 10 ml diluent was homogenised in a stomacher (AES Laboratoire, Combourg, France) for 2 min. From each surface sample one part of the diluent was used for microbiological analyses on the same day as sampling. The remaining part (approximately 7 ml) was collected and stored at − 18 °C until chemical analysis.

**Table 1:** Bacterial load of water samples, water disinfection and DWS disinfection

Broiler Farm	Type of water (number of samples)	Water contamination (CFU/ml)		Water disinfection during production				DWS disinfection during vacancy			
		TAC	PSEUDO	Frequency	Disinfection product	Active compound	Applied concentration ^a^	Frequency	Disinfection product	Active compound	Applied concentration
K1	Well water	> 300	> 300	2–3/week	DM CID (CID lines ^b^)	NaClO +KOH	0.00010%	1/round	CID 2000 (CID lines)	H_2_O_2_ +Acetic acid + Peracetic acid	0.050%
K2	Well water	> 300	20	/ ^c^	/	/	/	1/year	MS Oxy-Clean 1.0 (MS Schippers ^d^)	H_2_O_2_	Unknown
K3	Well water (2)	> 300	30 and > 300	Continuously	Di-O-Clean (MS Schippers)	ClO_2_	Week 1, 2, 3: 0.030% Week 4, 5, 6: 0.015%	1/round	Huwa-San Veterinary Applications (Sanac^e^)	H_2_O_2_	0.027%
K4	Well water	59	2	Daily	Top Clean Aqua (Topturn ^f^)	H_2_O_2_	0.20%	1/round	CID Clean (CID lines)	H_2_O_2_	1.0%
K5	Well water (2)	6 and 102	3 and 33	Week 1 and 2	CID Clean (CID lines)	H_2_O_2_	0.010%	1/round	CID Clean (CID lines)	H_2_O_2_	2.0%

Bacterial load of water samples taken at five broiler farms is represented as total aerobic count (TAC) and *Pseudomonas* count (PSEUDO). ^a^ Applied concentration of the disinfection product (not of the active compound); ^b^ CID lines, Ieper, Belgium; ^c^ /, No water disinfection applied; ^d^ MS Schippers, Arendonk, Belgium; ^e^ Sanac, Wervik, Belgium; ^f^ Topturn, Bergeijk, The Netherlands

### Microbiological characterisation of biofilm

#### Microbiological enumerations

For the surface samples, appropriate 10-fold dilutions were made in sterile 0,1% *w*/*v* Peptone Water with 0,85% *w/v* Salt (BioTrading, K110B009AA, Mijdrecht, The Netherlands) and spread plated. For the water samples, five times 1 ml was pour plated. Enumerations of total aerobic count (TAC) and *Pseudomonas* spp. were performed on both types of samples. The genus *Pseudomonas* is known to be abundantly present in natural waters [31, 32] and therefore probably also in the DWS of broilers. TAC was determined by plating on Plate Count Agar (PCA; Oxoid, CM0325) and incubating at 30 °C for three days based on the ISO 4833 standard. Presumptive *Pseudomonas* spp. were enumerated using Pseudomonas Agar Base (PAB; Oxoid, CM0559) with Pseudomonas CFC Selective Agar Supplement (Oxoid, SR0103) and incubation at 30 °C for two days (based on ISO 13720 standard) without oxidase confirmation test. The limit of quantification (LOQ) for microbiological enumerations was 1.00 log CFU/20cm^2^ for surface samples and one CFU/5 ml for water.

#### Isolate collection

It was already described that high levels of microorganisms originating from surfaces after C&D could be an indication for the presence of a biofilm [33]. Consequently, samples with counts of 2.00 log CFU/20cm^2^ or more after disinfection were considered as originating from potential biofilm carrying surfaces. From these samples, the dominant microbiota was collected for further identification. The plates with growth on the highest serial 10-fold dilutions represented the dominant microbiota. Based on morphology, 4 to 12 colonies were selected from PCA and 1 to 7 colonies were selected from PAB for each of the surface samples. Per water sample, 3 to 6 colonies and 1 to 3 colonies were selected from PCA and PAB, respectively. As for water samples some plates were overgrown whereby selection of dominant flora was difficult in those cases. Colonies were streaked on new PCA plates minimally three times to obtain pure cultures. The pure cultures were inoculated in Brain Heart Infusion (BHI; Oxoid, CM1135) with 15% glycerol (Merck, 8.18709.1000, Darmstadt, Germany), incubated for two days at 30 °C and kept at − 80 °C. From surface samples, a total of 241 isolates were collected from PCA and 105 from PAB. From the water samples, 22 and 10 isolates were collected from PCA and PAB, respectively. Collected isolates were classified as originating from samples in three bacterial quantity classes. For isolates collected from PCA, the class of less than 4 log CFU/20cm^2^ represented low bacterial numbers, the class of 4 to 7 log CFU/20cm^2^ represented medium numbers and the class of more than 7 log CFU/20cm^2^ represented high numbers. Isolates collected from PAB were classified as less than 4 log CFU/20cm^2^ (low numbers), 4 to 6 log CFU/20cm^2^ (medium numbers) or more than 6 log CFU/20cm^2^ (high numbers).

#### Isolate identification

From each isolate, except for those that could not be cultivated after storage at − 80 °C (38 out of 378 isolates), DNA was collected according to Strandén et al.(2003) [34]. Briefly, this included that pure cultures were first suspended in lysostaphin and incubated at 37 °C. Afterwards, proteinase K was added and incubation was performed at 60 °C for 10 min and at 100 °C for 5 min. DNA extracts were stored at 4 °C and used on the same day for (GTG)_5_ PCR based on Calliauw et al. (2016) [35] for clustering of the isolates. PCR amplifications were performed in an automated thermal cycler (GeneAmp® PCR System 9700, Applied Biosystems Europe, The Netherlands) with an initial denaturation (7 min at 95 °C) followed by 30 cycles of denaturation (1 min at 95 °C), annealing (1 min at 40 °C) and extension (8 min at 65 °C) and a final extension (16 min at 65 °C). PCR products were separated using the QIAxcel Advanced System (QIAGEN Benelux B.V., Venlo, The Netherlands) and QIAxcel DNA High Resolution Kit (QIAGEN Benelux B.V., 929,002) and clustering of the obtained fingerprints using BioNumerics version 7.6 software package (Applied Maths, Sint-Martens-Latem, Belgium) was performed according to Luyckx et al. (2016) [36]. Out of the 340 isolates included in the (GTG)_5_ fingerprint clusters, 200 were selected for identification based on the occurrence of their pattern and as representatives for visually defined clusters. For clusters with two or three isolates, one isolate (the middle one) was selected to identify the complete cluster. For clusters with four or more isolates, a minimum of two isolates were selected for identification. These were the outer isolates of the cluster possibly supplemented with an isolate in the middle to represent the largest possible diversity. The 16S rRNA gene was amplified for identification of the selected isolates using universal bacterial primers 16F27–1 (pA, 5′-3′ sequence: AGA GTT TGA TCC TGG CTC AG) and 16R1522 (pH, 5′-3′ sequence: AAG GAG GTG ATC CAG CCG CA), according to Brosius et al. (1978) [37]. The microbial DNA (± 25 ng/μl) was used as a template in the 50 μl PCR reaction containing 1x PCR buffer II (Applied Biosystems Europe, N8080153, The Netherlands), 1.5 mM MgCl_2_ (Applied Biosystems Europe, N8080153), 0.03 U AmpliTaq® DNA Polymerase (Applied Biosystems Europe, N8080153), 0.1 mM of each deoxynucleotide triphosphate (GE Healthcare Europe, GE28–4065-58, Diegem, Belgium) and 1.0 μM of the primers (Eurogentec, Seraing, Belgium). PCR amplifications were performed in an automated thermal cycler (GeneAmp® PCR System 9700, Applied Biosystems Europe) with an initial denaturation (1 min at 95 °C) followed by 30 cycles of denaturation (15 s at 95 °C), annealing (15 s at 63 °C) and extension (30s at 72 °C) and a final extension (8 min at 72 °C). PCR products were separated in the same way as for (GTG)_5_ PCR fragments except that method OM500 was used. In case no PCR product could be visualised, the annealing temperature during amplification was changed to 57 °C. When non-specific bands were amplified (visible as shorter or longer PCR products than the desired 16S gene of ±1500 bp), PCR reaction was performed again with bacterial primers 16F358 (*gamma, 5′-3′ sequence: CTC CTA CGG GAG GCA GCA GT) and 16R1485 (MH2, 5′-3′ sequence: TAC CTT GTT ACG ACT TCA CCC CA) providing a 1169 bp DNA fragment. PCR products were sequenced with forward and reverse primers by Macrogen Europe based on Sanger sequencing (Amsterdam, The Netherlands). Sequence reads of 500 bp or more were used for further analysis in EZtaxon [38]. The species in the database with the highest similarity (minimally 98.5%) and completeness was used to identify the isolates to the putative species level. When different species with the same similarity and completeness level occurred for an isolate, identification was performed to the genus level only. In total, 16S rRNA sequencing led to the identification of 191 of the 200 isolates. Together with the (GTG)_5_ fingerprint results, 330 out of 378 isolates could be identified to the genus or species level.

#### Evaluation of the biofilm-forming capacities of the isolates

The ability of a random selection of identified strains (*n* = 169) to form biofilms in polystyrene 96-well microtiter plates was determined based on Peeters et al. (2008) [39] with some modifications as described in the following section. Starting from an overnight liquid culture in Luria-Bertani broth (LB, Composition: 10 g l-1 trypton (Organotechnie, 19,553, La Courneuve, France), 5 g l-1 yeast extract (Organotechnie, 19,512), 10 g l-1 NaCl (VWR, 7647-14-5, Radnor, Pennsylvania) and 20 g l-1 glucose (Tereos Syral, 14,431–43-7, Marckolsheim, France)) at 30 °C, the turbidity of the overnight culture was compared to that of the positive control *Escherichia coli* MG 1655 to obtain a cell density of approximately 10^8^ CFU/mL. Subsequently a 1:100 dilution was made in LB. For each strain, 16 wells of a round-bottomed polystyrene 96-well microtiter plate (Greiner Bio-One, 650,101, Kremsmünster, Austria) were inoculated with 100 μl of this dilution. As negative control 16 wells were filled with sterile LB medium and as positive control 16 wells were filled with a 1:100 dilution of an overnight culture of *E. coli* MG 1655, which is a strong biofilm producer in this assay. The microtiter plate was incubated at 30 °C for 4 h to allow for the adhesion of the microorganisms. After this, the liquid (containing non-adhered cells) was removed by inverting the microtiter plate and all the wells were rinsed once with 100 μl of sterile ¼ Ringer’s solution (Biokar, BR00108, Beauvais, France). Fresh sterile LB medium was added to all wells and the microtiter plate was further incubated for 24 h at 30 °C. Subsequently, the liquid with culture was removed and all wells were washed three times with sterile ¼ Ringer’s solution to remove non-adhered cells. The remaining biofilm was fixated with 150 μl of 99% methanol (Acros Organics, 268,280,025, Geel, Belgium) per well for 15 min. After this the microtiter plate was emptied and air dried. Then, 100 μl of crystal violet solution used for Gram staining (Merck, 109,218, Darmstadt, Germany) was added to all wells for 20 min. The excess stain was removed by placing the microtiter plate under running tap water and washing was continued until the washings were free of the stain. Following, the microtiter plate was air dried for 2 h. Retained crystal violet was dissolved by adding 150 μl of 33% (*v*/v) glacial acetic acid (Merck, 100,063). The absorbance was measured at 590 nm using a microtiter plate reader (BioRad, 1,681,135, Hercules, CA, USA).

Based on the absorbance measured at 590 nm after crystal violet staining, biofilms were classified into following categories as previously described by Stepanović et al. (2000) [40]: non biofilm producer, weak, moderate or strong biofilm producer. The cut-off OD (ODc) was defined as three standard deviations above the mean absorbance of the negative control. Strains were classified as follows: ODstrain ≤ ODc = no biofilm producer, ODc < ODstrain ≤ (2 × ODc) = weak biofilm producer, (2 × ODc) < ODstrain ≤ (4 × ODc) = moderate biofilm producer and (4 × ODc) < ODstrain = strong biofilm producer.

### Chemical characterisation of biofilm

Chemical analyses were performed on all surface samples collected in the five broiler farms during the first sampling round (*n* = 43). Before chemical analyses, an extraction procedure was performed to separate the EPS from the microorganisms. This extraction procedure was first validated. This validation is described in the supplement 1 (Additional file 1) of this paper. Therefore, the remaining diluent part (¼ Ringer’s fraction after microbiological analyses) was sonicated (UP 400S, Hielscher, Germany) 3 times for 30s with an interval of 30s at an amplitude of 50% and a cycle of 0.5 in a water bath to disrupt the bacterial clumps. After centrifugation (Savant, SFA13K) at 13000 RCF for 10 min at room temperature, supernatant (containing EPS) was recovered and used for the chemical characterisation. Protein, carbohydrate and uronic acid analyses, which could be part of the biofilm’s EPS matrix, were performed as described by Maes et al. (2017) [33].

Briefly, proteins were quantified using Bradford Reagent (200/220 μL)(Sigma-Aldrich, B6916) and measurement of the OD595nm. Bovine serum albumin (BSA)(Sigma-Aldrich, A2153) was used as a standard. The quantification of carbohydrates was performed by adding 5% *w*/*v* phenol in water (30/230 μL) and concentrated H_2_SO_4_ (150/230 mL). After 5 min incubation at 90 °C and consequently 5 min at room temperature, the OD492nm was measured. Glucose (Sigma-Aldrich, G7528) was used as a standard. Uronic acid quantification was performed by adding sodium tetra borate in H_2_SO_4_, one hour incubation at 80 °C, four hours incubation in the dark and OD540nm measurement. Afterwards, 0.2% *w*/*v* m-hydroxydiphenyl (Sigma-Aldrich, 262,250) in H_2_SO_4_ with 2% *v*/v DMSO (Sigma-Aldrich, D8418) is added, followed by incubation in the dark at room temperature and the measurement of the OD540nm. D-galacturonic acid (Sigma-aldrich, 48,280) was used as a standard.

### Statistical analysis

All values of the chemical analyses are the result of the average of three technical replicates and all values of the biofilm-forming capacities are the result of the average of sixteen technical replicates. Statistical analyses on the obtained microbiological enumerations and chemical results were carried out using Statistical Analysis System software (SAS®, version 9.4, SAS Institute Inc., Cary, NC, USA). Distribution of the log transformed enumerations per microbiological parameter and quantification of the analysed chemical components was evaluated based on the histogram and QQ plot. For the representation of the contamination level, the values for microbiological and chemical analyses are represented by mean and standard deviation for normally distributed values. First quartile (Q1), median (Q2) and third quartile (Q3) are calculated for values that did not follow a normal distribution.

## Results

### Contamination of water samples on broiler farms

All broiler farms used well water as drinking water for the broiler chickens (Table 1). Water samples were taken at different broiler farms at the point just before entering the broiler house and before any disinfection product was administered. Table 1 shows the results of the bacterial load. TAC results varied from 6 to > 300 CFU/ml while enumerations for *Pseudomonas* spp. varied from 2 to > 300 CFU/ml. As during the very first sampling no water was sampled at K1 no isolates originating from water could be identified. The dominant microbiota of water samples from K2 collected from TAC were identified as *Bosea robiniae*, *Chryseobacterium scophthalmum* and *Rhizobium radiobacter* and the isolates collected from PAB were identified as *Delftia acidovorans* and *Pseudomonas peli*. *Chryseobacterium* spp., *Aeromonas media* and other *Aeromonas* spp. were identified as part of the dominant total aerobic water flora on K3, while *Aeromonas salmonicida* and *Pseudomonas koreensis* were identified among the isolates collected from PAB. The water flora on K4 contained predominantly *Arthrobacter russicus* and *Pseudomonas* spp. (isolated from TAC). The dominant flora of the other water sample taken on this farm mainly consisted of *Aeromonas* spp., *Bacillus* spp., *Chryseobacterium rhizosphaerae* and *Pseudomonas extremorientalis* isolated from TAC and *Pseudomonas granadensis* isolated from PAB. Disinfection products (mostly based on hydrogen peroxide or chlorine) were regularly applied in the drinking water in all farms during production, except in farm K2.

### Surface contamination in DWS of broiler houses

In total, 85 surfaces on the inside of the DWS were sampled after disinfection (Table 1). In all broiler farms, disinfection started by filling the drinking lines during vacancy with a disinfection product (always based on hydrogen peroxide as an active component). Afterwards, a rinsing step with water was performed. All farms performed this disinfection step after each production round, except for farm K2 where disinfection is only performed once a year. The applied concentration of the disinfection product varied per farm. Table 2 shows the results of the microbiological load and the quantification of the chemical biofilm components of the sampled surfaces. TAC results of all samples of the five farms varied between 1.87 and 9.00 log CFU/20cm^2^ while average and median values of samples taken at one sampling moment at one farm ranged from 4.27 to 7.19 log CFU/20cm^2^. Average TAC for all surfaces was 6.03 ± 1.53 log CFU/20cm^2^. Enumerations for *Pseudomonas* spp. were on average 0.88 log CFU/20cm^2^ lower than for TAC. Chemical analyses were performed on 43 of the 85 sampled surfaces. On 58% of the analysed surfaces, proteins were found. Carbohydrates and uronic acids were found only on 14 and 5% of the surface samples, respectively. When chemical and microbiological results were combined, 63% of the sampled surfaces during the first sampling round appeared to be contaminated with both microorganisms and at least one of the analysed chemical biofilm components.

**Table 2:** Presence of surface contamination in the DWS of broiler farms

Broiler Farm	Microbiological analysis			Chemical analysis							Presumable biofilms (%)
	n	Enumerations (log CFU/20cm^2^)		n	Proteins		Carbohydrates		Uronic acids		
		TAC	PSEUDO		% ^a^	Quantity (μg/20cm^2^)	%	Quantity (μg/20cm^2^)	%	Quantity (μg/20cm^2^)	
K1	8	5.53–**5.70** – 6.17	4.48–**4.89** – 5.26	8	25	15.70, 22.40 ^a^	0	/ ^b^	0	/	25
	15	7.19 ± 1.08	6.61 ± 1.12	0							
K2	6	6.28–**6.35** – 7.24	5.08–**6.29** – 7.42	6	83	14.00–**15.85** – 16.80	17	139.44 ^c^	0	/	83
K3	12	4.27 ± 0.64	3.36–**3.95** – 4.38	12	75	7.10–**16.95** – 19.05	17	59.63, 60.71 ^d^	17	28.40, 43.20 ^d^	83
	12	6.03–**6.97** – 7.63	5.32–**6.17** – 6.80	0							
K4	8	3.09–**4.55** – 4.68	1.92–**3.19** – 4.19	8	75	5.90–**18.40** – 26.85	12	64.32 ^c^	0	/	75
K5	9	4.30–**4.71** – 6.00	4.33 ± 0.72	9	33	0.00–**0.00** – 16.80	22	62.52, 94.04 ^d^	0	/	44
	15	7.13 ± 1.05	5.20–**5.67** – 6.11	0							

The number of sampled points (n) together with the proportion of quantifiable samples for chemical analysis (%) and values for total aerobic count (TAC), *Pseudomonas* count (PSEUDO), proteins, carbohydrates and uronic acids are shown for each farm sampling. Mean and standard deviation are given for values that are normally distributed. First quartile (Q1), median (Q2, in bold) and third quartile (Q3) are given for values that did not follow this distribution. Sampling points where both TAC (≥ 2 log CFU/20cm^2^) and one or several chemical components were quantified (>LOQ) are evaluated as presumably carrying biofilm. ^a^ %, proportion of quantifiable (>LOQ) samples given in percentage; ^b^ /, Values below LOQ; ^c^, Only one value obtained; ^d^, Only two values obtained

### Identification of microorganisms present on surfaces on the inside of the DWS of broiler houses

#### Isolates from PCA

**Fig. 1:**
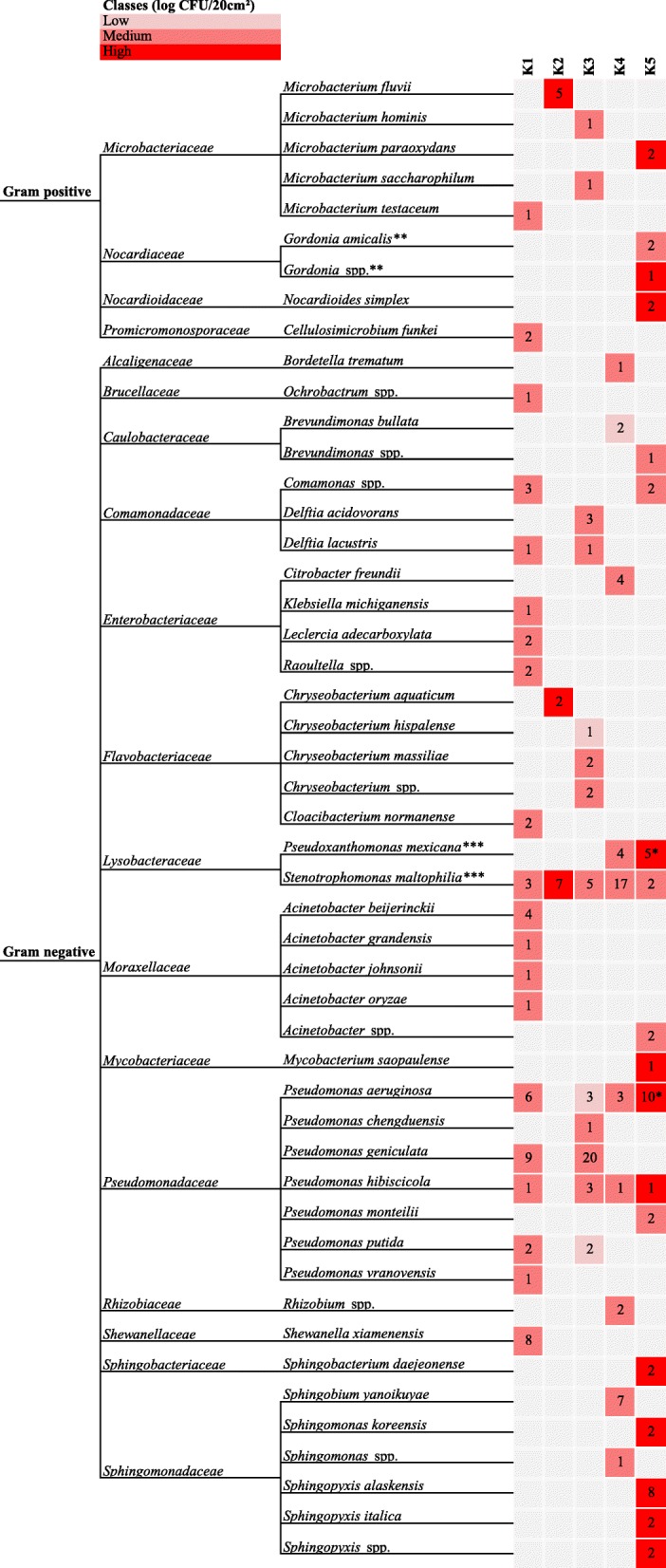
Identification of isolates from TAC of DWS samples. Family (based on http://www.bacterio.net/, verified on 25 January 2018), genus and species identity of isolates from PCA of inside surface samples of the DWS in five broiler farms (K1-K5) after disinfection. Different colors represent the magnitude of TAC enumerations of samples whereof the bacteria were isolated. * Indicates that the corresponding species was found during the first and second sampling round in the corresponding company. ** This species belongs to the family *Gordoniaceae* according to NCBI classification (verified on 27 January 2018). *** This species belongs to the family *Xanthomonadaceae* according to NCBI classification (verified on 27 January 2018). Classes (log CFU/20 cm^2^): Low < 4 log; Medium 4–7 log; High > 7 log

Among the few Gram positive isolates (*n* = 17; Fig. 1), the genus *Microbacterium* was identified in four of the sampled broiler farms. Each of the identified Gram positive species was found in only one of the sampled broiler farms and was present either in medium or high numbers. The identified Gram negative bacteria (*n* = 185) were mostly *Pseudomonas* (32.2% of the identified isolates) and *Stenotrophomonas* (16.8%)*,* and were found in four and five of the sampled farms, respectively. Moreover, concerning *Stenotrophomonas* it was the same species (*Stenotrophomonas maltophilia*) that occurred at all the broiler farms. *Pseudomonas aeruginosa* and *Pseudomonas hibiscicola* were found in four broiler farms (except for K2). However between farms there were differences in the most dominant microbiota. In broiler farm K1, besides *Pseudomonas* (36.5%), the genera *Shewanella* and *Acinetobacter* occurred with 15.4 and 13.5% of the isolates respectively. Only 14 isolates were collected in broiler farm K2, all belonging to the species *Stenotrophomonas maltophilia* (50.0%), *Microbacterium fluvii* (35.7%) or *Chryseobacterium aquaticum* (14.3%). *Pseudomonas* (64.4%), *Chryseobacterium* (11.1%) and *Stenotrophomonas* (species *maltophilia*, 11.1%) were the most identified genera at broiler farm K3, while the dominant microbiota at farm K4 mostly belonged to *Stenotrophomonas maltophilia* (40.5%) and *Sphingobium yanoikuyae* (16.7%). In broiler farm K5 the most dominant genera found were *Pseudomonas* (26.5%) and *Sphingomonas* (24.5%) and the species *Pseudoxanthomonas mexicana* (10.2%). Moreover, *Pseudomonas aeruginosa* and *Pseudoxanthomonas mexicana* isolates were found twice in time at farm K5.

#### Isolates from PAB

**Fig. 2:**
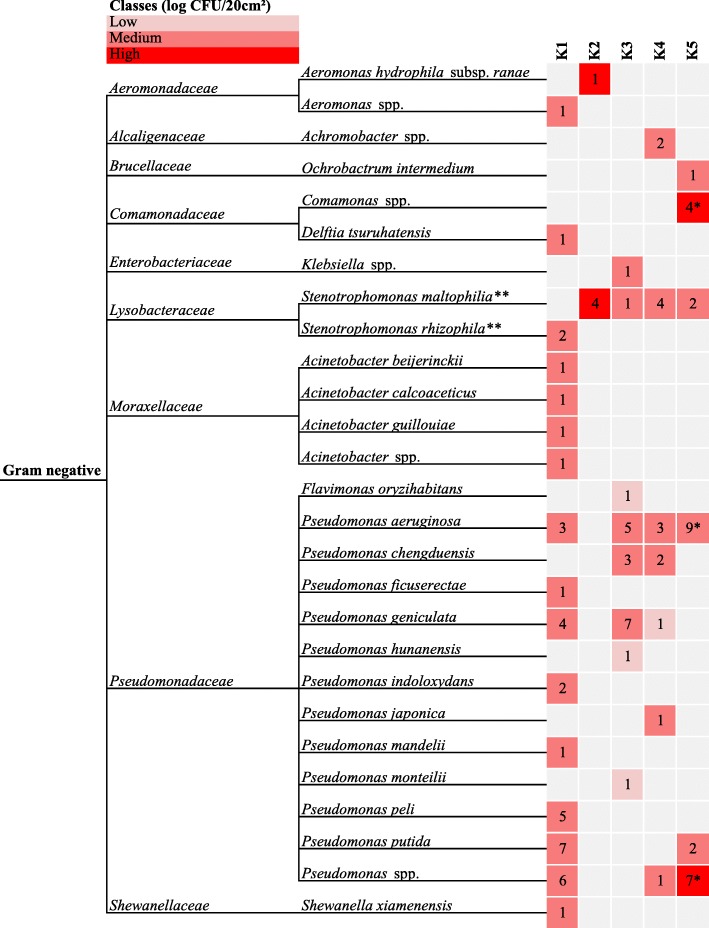
Identification of isolates from *Pseudomonas* spp. of DWS samples. Family (based on http://www.bacterio.net/, verified on 25 January 2018), genera and species identity of isolates from PAB of inside surface samples of DWS in 5 different broiler farms (K1-K5) after disinfection. Different colors represent the magnitude of PAB enumerations of samples whereof the bacteria were isolated. * Indicates that the corresponding species was found during the first and second sampling round in the corresponding company. ** This species belongs to the family *Xanthomonadaceae* according to NCBI classification (verified on 27 January 2018). Classes (log CFU/20 cm^2^): Low < 4 log; Medium 4–7 log; High > 7 log

The 102 identified isolates collected from PAB (Fig. 2) mostly belonged to the genera *Pseudomonas* (found on all farms but K2, 70.6% of the identified isolates) and *Stenotrophomonas* (found on all farms, 12.8%). More specifically in farm K1, the most identified species was *Pseudomonas putida* (18.4%). In broiler farm K2, 80% of the identified isolates were *Stenotrophomonas maltophilia*. *Pseudomonas geniculata* (35%) and *Pseudomonas aeruginosa* (25%) were the most identified species at farm K3, while the dominant microbiota at broiler farm K4 belonged to the species *Stenotrophomonas maltophilia* (28.6%) and *Pseudomonas aeruginosa* (21.4%). Also in broiler farm K5, the most common species found was *Pseudomonas aeruginosa* (36.0%) and moreover this species was found twice in time.

### Biofilm-forming capacities of microorganisms present on surfaces on the inside of the DWS of broiler houses

**Fig. 3:**
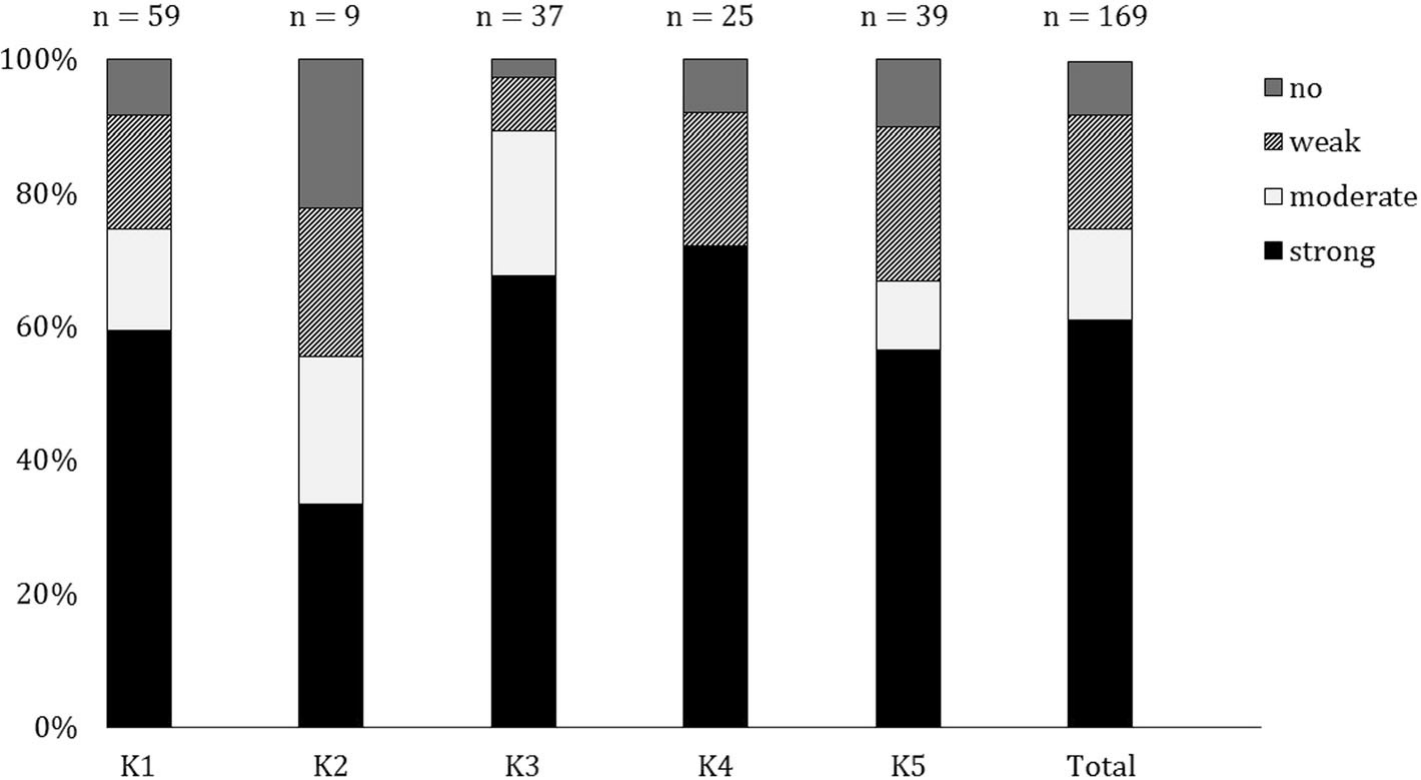
Prevalence of different classes of biofilm-formers in the broiler farms (%)

According to the classification of Stepanovic et al. (2000) [40], 92% of all tested microorganisms (*n* = 169) produced biofilm (Fig. 3), ranging from 78% of the tested isolates in farm K2 to 97% in farm K3. Of all the assessed isolates, 61% were strong biofilm producers, ranging from 33% in farm K2 to 72% in farm K4. Differences were observed between isolates from farm K2 (*n* = 9) and the other four farms in terms of a lower percentage of biofilm-forming isolates. The bacterial isolates per genus with strong biofilm-forming ability and their presence in the different farms (as strong biofilm producer) are summarised in Table 3. The strong biofilm producing bacteria mainly belonged to the genera *Pseudomonas* spp. and *Stenotrophomonas* spp. Of all the evaluated isolates of these genera, 83% (for *Pseudomonas*) and 87% (for *Stenotrophomonas*) were strong biofilm producers. Strong biofilm producers belonging to the genus *Pseudomonas* were found in every farm, except for K2. The genus *Stenotrophomonas* was present as strong biofilm producer on every farm. All isolates (with exception of two) belonging to the three most abundant species (i.e. *Pseudomonas aeruginosa*, *Pseudomonas geniculata* and *Stenotrophomonas maltophilia*) were evaluated as strong biofilm producers. *Pseudomonas aeruginosa* was present as a strong biofilm producer in every farm except for K2 and *Pseudomonas geniculata* was present as a strong biofilm producer in farms K1, K3 and K4. Other isolates with strong biofilm-forming capacities only occurred in one or two farms and belonged to the genera *Acinetobacter, Flavimonas, Nocardioides* and *Ochrobactrum*.

**Table 3:** Strong biofilm-forming genera and their presence on the different broiler farms

Identification	Evaluated isolates		TAC		PSEUDO		K1	K2	K3	K4	K5
	n	strong biofilm (%)	n	strong biofilm (%)	n	strong biofilm (%)					
*Pseudomonas* spp.	76	83	29	86	47	81	+ ^a^		+	+	+
*Stenotrophomonas* spp.	23	87	13	92	10	80	+	+	+	+	+
*Acinetobacter* spp.	9	44	5	20	4	75	+				
*Microbacterium* spp.	8	13	8	13					+		
*Delftia* spp.	5	40	4	50	1	0	+		+		
*Pseudoxanthomonas* spp.	4	25	4	25							+
*Shewanella* spp.	4	75	3	67	1	100	+				
*Comamonas* spp.	3	67	2	50	1	100					+
*Brevundimonas* spp.	2	50	2	50						+	
*Ochrobactrum* spp.	2	100	1	100	1	100	+				
*Sphingobium* spp.	2	50	2	50						+	
*Sphingomonas* spp.	2	50	2	50							+
*Flavimonas* spp.	1	100			1	100			+		
*Nocardioides* spp*.*	1	100	1	100							+

The number of evaluated isolates (n) together with the proportion of strong biofilm-formers given in percentage (%) is shown per genus. ^a^ +, indicates the presence of the genus as strong biofilm former in the corresponding broiler farm

## Discussion

### Contamination of water samples on broiler farms

Microbiological load of the incoming water samples (without disinfection product) ranged from 6 to > 300 CFU/ml. This was generally lower than reported by Maharjan (2016) [41]. Participating broiler farms used water disinfection products based on oxidising agents such as chlorine or hydrogen peroxide to control microbial growth. However, the used concentrations are lower than recommended by the suppliers and consequently a sufficient reduction of the microbial level is not guaranteed.

Identified dominant microbiota in water samples were unique per farm and consisted mostly of Gram negative bacteria. None of the species identified in the incoming water were also found as dominant flora on the inside of the DWS. On genus level on the other hand, the genus *Pseudomonas* (one of the most identified genera on the inside of the DWS) also occurred in all the water samples. Also *Chryseobacterium* spp. were found in three of the five water samples. The identified microorganisms from water samples are generally not involved in disease development in poultry [42]. However, *Aeromonas media*, is reported as a putative human pathogen [43].

### Surface contamination in DWS of broiler houses

In all the broiler farms, disinfection of the DWS was performed during vacancy with oxidising agents, containing at least hydrogen peroxide. Despite regular disinfection, most sampled surfaces on the inside of the DWS of the broiler houses showed high microbiological counts. No peer reviewed results could be found concerning microbiological contamination on similar surfaces. However SE Watkins found comparable counts (6.35 and 6.83 log CFU/sponge; personal communication, December 19, 2017, University of Arkansas) to this study on surfaces on the inside of the drinking water system such as the end of the lines. These high numbers of microorganisms are possibly due to the insufficiently high concentrations at which the disinfectants were applied. Also, disinfection is not preceded by a cleaning step (which would loosen and eliminate organic materials) whereby the present microorganisms are not reached and affected sufficiently. It is well known that cleaning of DWS is not obvious since it is a mostly closed system and applying the needed mechanical force is not evident. Beside, the survival can be caused by antioxidant strategies, resistance to the disinfectant, the structure of the microbial biofilm communities (which causes reduced diffusion of the active components) and many other defensive strategies of microorganisms whether or not present in biofilms [44]. Taking all these aspects into account, it is better to speak of DWS sanitation instead of disinfection.

No previous studies were found where surface samples of DWS on primary production farms were chemically analysed for biofilm EPS components. In this study, 63% of the samples where chemical analysis was performed contained at least one of the chemical components (proteins, carbohydrates or uronic acids). The presence of high numbers of microorganisms in combination with chemical components (possibly originating from EPS or organic pollution) sampled after the application of disinfectants on the surface can be an indication for the presence of a biofilm [33]. This means that in this study, 63% of the analysed surfaces would be identified as carrying biofilm. This is a much higher number compared to surfaces in the food industry where the presence of biofilm (determined in the same way as in the current study) was suspected in 17% of the cases [33].

### Characterisation of isolates collected from the DWS of broiler houses

To our knowledge, no previous studies were performed describing the identity and biofilm-forming capacity of microorganisms isolated from the inside of the DWS in broiler farms. Overall, Gram negative bacteria were identified to a higher extent compared to Gram positive bacteria. This is possibly due to the fact that Gram negative bacteria are generally better biofilm formers and that the niche in DWS is more favourable [45, 46]. The dominant bacteria identified over the participating farms were largely similar except for K2. On this farm, except for *Stenotrophomonas maltophilia*, the dominant flora differed from the other farms. Besides a smaller sample size and consequently less collected isolates, on farm K2, water disinfection was not applied and DWS disinfection only took place once a year. There is an indication that performing less frequent disinfection leads to a smaller diversity of the microbial flora on inside surfaces of the DWS. However, due to the low number of isolates and the single case of the specific character of farm K2, it is difficult to draw general conclusions. Bacteria originating from surfaces on the inside of the DWS (both with or without detection of chemical components) in the other four broiler farms and collected from TAC (but also identified on PAB) mainly belonged to the species *Stenotrophomonas maltophilia* (17% of the identified isolates), *Pseudomonas geniculata* (14%) and *Pseudomonas aeruginosa* (11%). Species that were also abundant but not identified on PAB were *Pseudoxanthomonas mexicana*, *Sphingopyxis alaskensis* and *Shewanella xiamenensis* (all 4% of the identified isolates from TAC). According to Anzai et al. (2000) [47], *Pseudomonas beteli, Pseudomonas geniculata* and *Pseudomonas hibiscicola* should not be included in the genus of *Pseudomonas* (sensu stricto) because of a higher level of homology (99.2–99.5%) with *Stenotrophomonas maltophilia* based on the 16S rRNA gene sequence. Although further extensive studies are required for definite taxonomic conclusion, this would shift the prevalence of *Stenotrophomonas maltophilia* to 34%. Rożej et al. (2015) [48] reported the abundance of *Stenotrophomonas maltophilia* and *Pseudomonas aeruginosa* in a model for drinking water distribution systems. According to these authors the abundance of these microorganisms was due to the high ability to settle and multiply on the surface of plastic pipes. Moreover, these two species have previously been found in water supply networks for human use such as homes, schools and hospitals [49, 50]. *Pseudomonas aeruginosa* is a versatile Gram negative bacterium that is one of the top three causes of opportunistic human infections [51] that may become multidrug resistant [52]. Moreover, different studies reported the high mortality rate in broiler chicks due to *Pseudomonas aeruginosa* infection [53–55]. *Stenotrophomonas maltophilia* is an environmental global emerging multidrug resistant microorganism that is most commonly associated with respiratory infections in humans [56]. *Pseudomonas aeruginosa* and *Stenotrophomonas maltophilia* are frequently co-isolated from lungs of cystic fibrosis patients and evidence suggests that *Stenotrophomonas maltophilia* modulates the virulence of *Pseudomonas aeruginosa* in a multispecies biofilm [57]. Although *Stenotrophomonas maltophilia* was detected in the caecal content of broiler chickens [58] and chicken eggs [59], no link with water quality and with disease development in broiler chicks associated with *Stenotrophomonas maltophilia* was reported*.*

In this study 92% of all tested isolates had the ability to produce biofilm and 61% were even strong biofilm producers. Remarkably, a much lower percentage of isolates collected on farm K2 were evaluated as strong biofilm formers compared to the other broiler farms. This might indicate that performing more frequent disinfection of the DWS results in the presence of more strong biofilm-forming microorganisms. Again, due to the low number of tested isolates it is however difficult to draw general conclusions. Almost all the isolates belonging to the three most abundant species (*Stenotrophomonas maltophilia*, *Pseudomonas geniculata* and *Pseudomonas aeruginosa*) were strong biofilm producers. When applying the taxonomic classification of Anzai et al. (2000) [47], the percentage of strong biofilm producing bacteria would shift from 61% *Pseudomonas* and 19% *Stenotrophomonas* to 40% *Pseudomonas* and 43% *Stenotrophomonas*. There must be taken into account that the used assay to evaluate biofilm forming potential had his limits. Notwithstanding using a positive control and the normalization of the cultures by OD, CFU densities vary between different organisms and no corrections were made for differences in growth rates and effective cell numbers.

Zoonotic pathogens mostly associated with poultry [60], such as *Campylobacter* spp. and *Salmonella* spp., were not identified among the dominant microbiota of water and DWS surface samples. This is because if these pathogens are present, this would be in such low number that they would not be identified among the dominant flora on TAC. Detection methods for *Campylobacter* spp. and *Salmonella* spp. were performed on samples collected in three out of the five participating broiler farms during the second sampling round. Although no *Campylobacter* spp. or *Salmonella* spp. were identified in these samples, the presence of biofilm-forming bacteria present in DWS could be a potential risk for the protection of these pathogens. The survival of culturable *Campylobacter jejuni* increased when cultured with a biofilm of a community sampled from a water drinker in a poultry house in a study by Hanning et al. (2008) [61]. The sampled community consisted mainly of *Pseudomonas* spp., *Staphylococcus* spp.*, E. coli, Bacillus* spp. and *Flavobacterium* spp. Culotti and Packman (2015) [62] reported not only a prolongation of the survival of *Campylobacter jejuni* when co-cultured with *Pseudomonas aeruginosa* under aerobic conditions, but also an enabling of the growth of *Campylobacter jejuni* on the surface of *Pseudomonas aeruginosa* biofilms. Comparable results were reported in other studies [18, 63]. The presence of *Pseudomonas* spp. could also favour the growth of *Salmonella* in biofilms. The biovolume of dual-species biofilms of *Salmonella* and *Pseudomonas* spp. increased 3.2-fold compared to single-species biofilms of *Salmonella* [64]. However, knowledge is lacking about the importance of biofilm or strong biofilm formers in the protection of zoonotic pathogens in practice. The results in this study will be the basis for more research of our group in a broiler farm biofilm model system concerning the interaction between *Salmonella* spp. and the obtained field isolates from the DWS.

## Conclusions

Despite regular sanitation with oxidising disinfection products, sampled surfaces on the inside of DWS in broiler houses showed rather high (average of 6.03 ± 1.53 log CFU/20cm^2^) microbiological counts. Also, 63% of the sampled surfaces contained at least one of the analysed chemical components. The presence of high numbers of microorganisms in combination with chemical components is indicative for the presence of biofilm.

The most identified species over the five sampled broiler houses were *Stenotrophomonas maltophilia*, *Pseudomonas geniculata* and *Pseudomonas aeruginosa*. Moreover, these species were in this study identified as strong biofilm formers. It is also known that some of these microorganisms can cause disease and death in humans and chickens, whereby they are important to monitor and eliminate.

Even without *Salmonella* spp. and *Campylobacter* spp. being detected in the present study, it was already shown in lab studies that biofilm could play a role in the maintenance of these pathogens in the drinking water system of broiler chickens. More research will be done in a biofilm model system concerning the interaction between these pathogens and microorganisms originating from DWS.

## Additional file

Additional file 1: The validation of the extraction procedure used to separate the EPS from the microorganisms in the sampled biofilms. (DOCX 19 kb)
