# Supplementary material for: Occurrence and characterisation of biofilms in drinking water systems of broiler houses
Source: BMC Microbiol. 2019 Apr 15;19:77. doi: 10.1186/s12866-019-1451-5 (PMC6466764; doi:10.1186/s12866-019-1451-5)
Supplement: Supplementary file 1 — The validation of the extraction procedure used to separate the EPS from the microorganisms in the sampled biofilms. (DOCX 19 kb) [file 12866_2019_1451_MOESM1_ESM.docx]

**Supplementary information on the EPS characterization assay**

Introduction

Before starting the experiment as described in the paper/study, several extraction methods were tested for extracting the EPS i.e. sonication, extraction with an acid solution or extraction using a salt solution. Pressure differences created by sonication, followed by centrifugation allows the EPS to go into the solution. This extraction method is often used and the chances to have cytoplasmatic contamination is reduced to a minimum in comparison with chemical extraction methods (Comte et al., 2007; More et al., 2014). Extraction with an acid solution is based on the fact that the treatment of the biofilm with the acid disturbs the interaction and strengthens the repellent forces between the bacteria and the EPS. Acid treatment can lead to disruption of the cell integrity and thus cell lysis (More et al., 2014; Sheng et al., 2010). Another option is to extract the EPS using a salt solution. This method is based on the fact that the surface of the bacterial cell membranes and cell walls have positive and negative charged groups. The EPS also has positive and negative charged zones, leading to ionic interactions between the cell surface of the bacteria and the EPS. By using salt with a high ionic strength, the ionic interactions between the bacteria and the EPS will be broken. As a result, EPS will get free (Chiba et al., 2014). The use of salt solution is seen as a mild but effective method for the extraction of EPS (More et al., 2014).

The aim of this experiment was to test the three described extraction methods in their efficiency to extract EPS so the EPS composition can be determined.

Materials and methods

Three different EPS extraction methods: sonication, 0.2M H_2_SO_4_ and 1.5M NaCl were tested. Therefore, *Staphylococcus aureus (LMG8224)*, *Pediococcus pentosaceus (LMG11488)* and *Leuconostoc mesenteroides (LMG6908)* were grown to form biofilms in 96-well plates. First, *S. aureus, P. pentosaceus* and *L. mesenteroides* were grown in optimal growth media i.e. TSB and MRS for respectively *S. aureus* and the lactic acid bacteria. After 22h incubation under shaking conditions at their optimal growth temperature (37, 30 and 30°C respectively for *S. aureus, P. pentosaceus* and *L. mesenteroides*), cells were obtained by centrifugation (10min, 4°C, 4000g) and washed twice with sterile physiological water (0.9 % NaCl). The cell pellet was resuspended in TSB + 1% glucose when *S. aureus* was used, and in MRS for *P. pentosaceus* and *L. mesenteroides,* so that an initial amount of 10^7^CFU/ml was reached*.* Then, 200µl of the cell suspensions were transferred in the wells of a 96-well plate and incubated for 24h at their respective optimal growth temperature. After incubation, the medium was gently removed and the wells were washed twice with 200µL of sterile physiological water (0.9 % NaCl). The biofilm was scraped from the wells with a sterile pipette tip and suspended in 200 µL of sterile physiological water (0.9 % NaCl). These suspensions were used for the EPS matrix extractions. Extraction methods were performed as followed:

Sonication: The suspensions (in Eppendorf tubes) were placed in a sonication bath for 3 times 30s at 50% amplitude and 0.5 cycle. Afterwards the suspensions were centrifuged (10min, 5000g) and the supernatant was used for the chemical characterization.

0.2M H_2_SO_4_: 1M H_2_SO_4_ was added to the suspensions in a 1/5 ratio to get a final concentration of 0.2M H_2_SO_4_. The suspensions were vortexed and a glass bead was added before they were incubated (3h, shaking 300 RPM, 4°C). Afterwards the suspensions were centrifuged (20min, 12500g) and the supernatant was used for the chemical characterization.

1.5M NaCl: 3M NaCl was added to the suspensions in a ½ ratio to get a final concentration of 1.5M NaCl. The samples were vortexed and centrifuged (10min, 5000g). The supernatant was used for the chemical characterization.

Results

The results of the protein, carbohydrate and uronic acid content obtained after extraction of the EPS is presented in Table 1.

Table 1: Chemical characterization of the EPS of biofilms of *Staphylococcus aureus*, *Leuconostoc mesenteroides* en *Pediococcus pentosaceus* extracted with different EPS extraction methods. DL = Detection Limit, n = 3

| **Extraction method** | **Proteins**  **(µg/mL)** | **Carbohydrates**  **(µmol/mL)** | **Uronic acids**  **(µg/mL)** |
| --- | --- | --- | --- |
| *Staphylococcus aureus* | | | |
| Sonication | 495 ± 0.004 | 0.64 ± 0.05 | 7.56 ± 0.001 |
| 0,2M H_2_SO_4_ | 246 ± 0.003 | 0.50 ± 0.04 | 3.11 |
| 1,5M NaCl | 335 ± 0.001 | 0.28 ± 0.02 | 2.56 ± 0.002 |
| *Leuconostoc mesenteroides* | | | |
| Sonication | 267 ± 0.001 | 0.071 ± 0.01 | < DL |
| 0,2M H_2_SO_4_ | 248 ± 0.004 | 0.113 ± 0.01 | < DL |
| 1,5M NaCl | 265 ± 0.004 | 0.158 ± 0.08 | < DL |
| *Pediococcus pentosaceus* | | | |
| Sonication | 266 ± 0.001 | 0.34 ± 0.02 | 1.44 ± 0.001 |
| 0.2M H_2_SO_4_ | 249 ± 0.002 | 0.58 ± 0.09 | < DL |
| 1.5M NaCl | 260 ± 0.001 | 0.14 ± 0.04 | < DL |

The extraction efficiency of the EPS derived from the biofilm of *S. aureus* was the highest when using sonication. The obtained concentrations of proteins, carbohydrates and uronic acids are clearly higher compared to the ones obtained with 0.2M H_2_SO_4_ and 1.5M NaCl. Regarding the biofilms of *L. mesenteroides*, also here the highest concentrations of proteins were obtained after sonication, however a higher extraction of carbohydrates was obtained using a salt solution. In none of the extraction methods, we were able to detect uronic acids in the *L. mesenteroides* biofilms. For the *P. pentosaceus* biofilms, sonication resulted in the highest protein concentration, and detectable concentrations of uronic acids, while the highest carbohydrate content was measured after acid based extraction. From these results it can be concluded that the amount of carbohydrates extracted is variable, depending on the extraction method used, and the microorganism used for biofilm formation. For proteins and uronic acids, sonication gives the highest extraction efficiency.

Therefore sonication was chosen to use in further EPS studies, as it includes fewer steps for extraction and it will not interfere with the components as chemical extraction can do (e.g. foam formation when high salt concentrations are used in a protein rich environment).

References

More, T.T., Yadav, J.S.S., Tyagi, r.D., Surumpallli, R.Y. (2014). Extracellular polymeric substances and their potential environmental applications. Journal of Environmental Management, 144, 1-25.

Chiba, A., Sugimoto, S., Sato, F., Hori, S., Mizunoe, Y. (2014). A refined technique for the extraction of extracellular matrices from bacterial biofilms and its applicability. Microbial Biotechnology, 10, 1-12

Sheng, G.P., Yu, H.Q., Li, X.Y. (2010). Extracellular polymeric substances (EPS) or microbial aggregates in biological wastewater treatment systems: a review. Biotechnology Advances, 28, 882-894.

Comte, S., Guibaud, G., Baudu, M. (2007). Effect of extraction method on EPS from activated sludge : an HPSEC investigation. Journal of Hazardous Materials, 140, 799-806.
